# Validation of the Simplified Chinese Version of the Brief Diabetes Quality of Life (DQoL) Questionnaire Based on a Cross-Sectional Study

**DOI:** 10.3390/ijerph17238792

**Published:** 2020-11-26

**Authors:** Zhijia Tang, Xinying Jiang, Lan Hong, Zhen Feng, Qingfeng He, Jing Yuan, Xiaoqiang Xiang

**Affiliations:** Department of Clinical Pharmacy and Pharmacy Administration, School of Pharmacy, Fudan University, Shanghai 201203, China; zjtang@fudan.edu.cn (Z.T.); zjjxyhao@hotmail.com (X.J.); honglan@fudan.edu.cn (L.H.); 20211030065@fudan.edu.cn (Z.F.); qf_he@fudan.edu.cn (Q.H.); jyuan@fudan.edu.cn (J.Y.)

**Keywords:** quality of life, questionnaire, diabetes mellitus, validity, Chinese

## Abstract

(1) Objective: To assess the reliability and validity of the simplified Chinese version of the brief Diabetes Quality of Life (DQoL) questionnaire in measuring health-related quality of life (HRQoL) in Chinese type 2 diabetes (T2D) patients. (2) Methods: A cross-sectional validation study including 277 patients was conducted at a tertiary hospital in Shanghai, China during April–May, 2018. The English brief DQoL was forward and back-translated into simplified Chinese. The expert interview, exploratory factor analysis (EFA), and Spearman correlation with the 5-level version of EuroQoL-5 (EQ-5D-5L) were employed to establish its validity. The internal reliability was assessed by Cronbach’s alpha. Participants were also stratified into subgroups to evaluate if the Chinese brief DQoL had more test effectiveness in a specific subpopulation. (3) Results: No items were removed from the original English brief DQoL based on the results of factor analysis and expert interview. The Spearman coefficient revealed a low-moderate inverse correlation between DQoL and EQ-5D-5L index and visual analogue scale (VAS), respectively (ρ1 = −0.364, *p* < 0.0001; ρ2 = −0.514, *p* < 0.0001). The Cronbach’s alpha coefficient of the final scale was 0.731. (4) Conclusions: The simplified Chinese version of the brief DQoL questionnaire showed reasonable reliability and validity, suggesting its potential appropriateness for evaluating quality of life in Chinese T2D patients. More future efforts should be made to generalize the application of the findings.

## 1. Introduction

Diabetes is a chronic disease with serious short-term and long-term consequences [1]. In recent decades, the global prevalence of diabetes increased greatly. In China, there were 116 million people aged 20–79 years with diabetes in 2019, which ranked top of the world and was expected to grow by 21% in the next 10 years [2]. The significant increase is mostly attributed to type 2 diabetes. According to the World Health Organization (WHO), type 2 diabetes (T2D) presents as a spectrum of metabolic abnormalities in glucose, lipid and protein and is responsible for over 90% of all diabetes cases worldwide. T2D puts a burden on both patients themselves and the healthcare system. Clinical and patients’ perceived outcomes are gradually recognized as equally important in assessing the impact of treatment, particularly for chronic diseases like T2D. Diabetes therapy, macrovascular (i.e., cardiovascular and cerebrovascular conditions) and microvascular (i.e., neuropathy, nephropathy, ocular lesions and foot disease) complications, as well as the trouble of taking oral antidiabetic drugs (OADs) several times a day, the fear of insulin injection, and hypoglycemic events bring inconvenience to patients’ lives and impair their quality of life [3].

Although disease control can be achieved by technology and modern medicine, the ultimate goal of diabetes care is to enhance patients’ quality of life which focuses on their emotional feelings and the psychological impact of the illness and its treatments on daily life. Health-related quality of life (HRQoL) is a multi-dimensional concept that includes physical, psychological and social aspects of personal health and is one of the most widely used measures to subjectively evaluate the health impact of disease management. The relationship between T2D and reduced HRQoL has long been confirmed [4]. Previous studies showed that age, body mass index (BMI), duration of disease, glucose level, hypertension, depression, complications, comorbidities, physical activity, diet, and glucose check frequency were risk factors associated with HRQoL in T2D patients [5,6].

There are many different scales to describe HRQoL. The generic instrument EuroQoL-5 (EQ-5D) has been recommended by the National Institute for Health and Clinical Excellence (NICE) to assess HRQoL [7,8]. It is brief and easy to use but may not accurately reflect the true scores in patients with specific disease. The original version of EQ-5D (EQ-5D-3L) was introduced in 1990 which comprises 5 dimensions (mobility, self-care, usual activities, pain/discomfort, and anxiety/depression) and each dimension has 3 levels (no problems, some problems, and extreme problems) [9]. In 2005, a new 5-level version of EQ-5D called the EQ-5D-5L was released. The EQ-5D-5L comprises the same 5 dimensions as EQ-5D-3L but each dimension now has 5 levels (no problems, slight problems, moderate problems, severe problems, and extreme problems) [9]. The EQ-5D-5L defines a total of 3125 possible health states and shows increased reliability and sensitivity while maintaining feasibility and potentially reducing ceiling effects [9]. It also shows more discrimination in patients with T2D than EQ-5D-3L [10,11].

The disease-specific instruments are used according to a disease’s characteristics and symptoms and, therefore, are more sensitive in the target population. The Diabetes Quality of Life (DQoL) questionnaire, a diabetes-specific instrument, was considered to be amenable for promoting the provider-patient communication on diabetes treatment. Since its full form of 42 items is too lengthy to be completed as part of a provider’s routine office visit, researchers developed a brief DQoL comprising 15 questions in American patients in 2004 [12]. This brief version of DQoL demonstrated an equal reliability and validity in assessing HRQoL and disease control and only takes up to 10 min to complete [13]. It serves as a tool for quickly screening patients for specific treatment-related problems that predicts self-reported diabetes care behaviors and satisfaction with diabetes control, as effectively as the full version of the instrument [12]. However, it is only available in English and has not been validated for use with Chinese patients. A 24-item short version of the Chinese DQoL was developed in 2018 but the validity still needs to be further examined to optimize the structure [14].

This study aims to evaluate the reliability and validity of the simplified Chinese version of the 15-item brief DQoL by comparing it to EQ-5D-5L, in order to provide an effective vehicle for evaluating the HRQoL for Chinese T2D patients.

## 2. Methods

This was a cross-sectional questionnaire validation study with aim to develop the simplified Chinese version of the brief DQoL questionnaire. The study was conducted in two phases: a small sample pilot test, and a formal test. All participants were notified of the study purpose and provided informed consent before enrollment. The study was reviewed and approved by the Ethics Committee of Minhang Hospital (No. k2019-029).

### 2.1. Instrument Selection and Adaptation

Through a literature review, the 15-item brief version of DQoL was used in this study to obtain the information from patients including satisfaction with treatment, impact of treatment, worry about the future effects of diabetes, and worry about social/vocational issues [12]. The items were scored on a 5-point Likert scale, from 1 (never) to 5 (all the time) and from 1 (very satisfied) to 5 (very dissatisfied), respectively. Lower scores can be interpreted as less problem frequency or satisfaction, and therefore indicate better quality of life [12]. The brief DQoL was forward- and back-translated from the original English language into simplified Chinese and reviewed by two bilingual researchers independently for conceptual and item equivalence [15]. Thereafter, an additional three people with excellent knowledge of English but unfamiliar with diabetes or DQoL were invited to compare the accuracy of the translation with source language.

The Chinese version of EQ-5D-5L was employed as a reference to examine the criterion-related validity. In the first part of EQ-5D-5L, a descriptive health state profile was collected and transferred into a summary index score using a standard Chinese specific EQ-5D-5L value set [16]. The index score ranged from 0 (dead) to 1 (full health), with higher scores indicating higher health. In the second part, patients were asked to mark on a visual analogue scale (VAS) which rated their perceived health from 0 (the worst imaginable health) to 100 (the best imaginable health) [9].

### 2.2. Pilot Test

In the pilot test, the draft DQoL along with the original English version underwent content validation by two endocrinologists and two pharmacists, who held Master’s or above degrees and had at least two years of experience studying abroad. The draft DQoL was also distributed to a small group of patients at Minhang Hospital affiliated to Fudan University in Shanghai, China, in order to ensure the easy understanding and appropriateness of the language to laypersons. These patients were selected by convenience and their data were not included in subsequent statistical analysis. Inclusion criteria were: (1) diagnosis of T2D by the 1999 WHO criteria [17], with or without comorbidity confirmed by means of interview or electronic health record (EHR); (2) 18 years of age or older; (3) normal cognition; and (4) gave voluntary, signed informed consent. Exclusion criteria included severe diabetes-unrelated organ damage, cognitive impairment, dementia, current psychiatric disorders, and unwillingness or inability to respond to questions. In accordance with the results of validation tests from the expert panel and patients, the draft DQoL was revised and a final version was completed.

### 2.3. Formal Test

In this phase, participants were recruited according to the same inclusion and exclusion criteria used in the pilot study through voluntary and convenience sampling at Minhang Hospital during April–May, 2018. The sample size was calculated based on item-subject ratio where better outcomes usually occur with larger sample size. Based on the widely accepted rule of thumb, a ratio of 1:10 was used in this study [18]. Therefore, the minimum required sample size was set at 150 in order to support development of a stable questionnaire, as well as to have sufficient power (α = 0.05; two-tailed) to detect a statistically significant group difference among target population. Each participant was given the final version of Chinese brief DQoL and EQ-5D-5L during office visits and encouraged to finish independently to minimize the influence from family members. Assistance (i.e., reading the questions) was offered to participants who cannot fill the questionnaire by themselves. Demographical data including age, gender, BMI, education level, occupation, duration of disease, the most recent fasting plasma glucose (FPG), diabetes complications, and comorbidities were collected from interview and health information system (HIS) at Minhang Hospital.

### 2.4. Data Analysis

#### 2.4.1. Descriptive Statistics

All data were screened by two researchers independently and imported into Excel 2013 (Microsoft Corporation, Redmond, WA, USA). Incomplete and wrong (i.e., selected more than one answer in any questions) questionnaires were excluded. Descriptive statistics were used to summarize the demographic data and scores of Chinese brief DQoL and EQ-5D-5L from both the overall and subgroup perspectives. Either an independent t test or nonparametric (Mann–Whitney U or Kruskal–Wallis) test was used to determine the differences between mean scores for subgroups according to the normality results.

#### 2.4.2. Validity

In pilot test, content validity was established by the expert interview. During the formal test, two measures of validity were conducted, one for construct validity (exploratory factor analysis) and one for criterion-related validity (Spearman correlation). A correlation coefficient between 0.3 and 0.5 (−0.3 and −0.5) was interpreted as a low correlation while 0.5 to 0.7 (−0.5 to −0.7) as a moderate correlation, 0.7 to 0.9 (−0.7 to −0.9) as a high correlation, and greater than 0.9 (lower than −0.9) as a very high correlation [19].

#### 2.4.3. Reliability

The reliability of Chinese brief DQoL was assessed using Cronbach alpha coefficient as a measure of internal consistency in which greater than 0.7 was considered acceptable [18].

All data were analyzed with IBM (Armonk, NY, USA) SPSS Statistics for Windows, Version 20.0. Statistical significance was defined as *p* < 0.05.

## 3. Results

### 3.1. Participants Characteristics

After eliminating the incomplete and wrong ones, we recovered 277 questionnaires. Mean age was 64.0 years old (standard deviation [SD] = 9.7) and 57.4% were male. Mean BMI was 24.60 kg/m^2^ ([SD] = 3.78). Majority of the patients had a secondary degree or below (71.8%), and were retired (84.5%). Mean duration of disease was 12.3 years ([SD] = 8.0). For laboratory indicators, mean FPG was 8.48 mmol/L ([SD] = 6.37). For diabetes complications and comorbidities, 55.6% and 22.7% of participants had at least one complications or comorbidities, respectively. The sociodemographic and clinical characteristics of the participants are shown in Table 1.

The mean score of the Chinese brief DQoL, EQ-5D-5L index, and EQ-VAS was 28.93 (standard deviation [SD] = 5.974), 0.904 ([SD] = 0.132), and 72.36 ([SD] = 13.961), respectively. Participants were stratified into subgroups based on whether they had risk factors (i.e., high BMI, old age, long disease duration, and high FPG). As shown in Supplementary Table S1, for each independent variable, the distribution of scores for both subgroups significantly deviated from a normal distribution while they had similar shape assessed by visual inspection. Therefore, the Mann–Whitney U test and Kruskal–Wallis test were chosen to determine if there were differences in scores between subgroups (Table 2). The score of Chinese brief DQoL was statistically significantly higher in participants with longer disease duration (≥10 years) than the short duration group (*p* = 0.001). The score of EQ-5D-5L was significantly lower among older participants (≥60 years) and with longer disease duration (≥10 years) (*p* < 0.05). In particular, participants with FPG ≥ 7 mmol/L also had a significantly lower EQ-VAS score than those whose glucose were better controlled (*p* = 0.012), as predicted, which indicated worse HRQoL. Both scales showed that HRQoL decreased significantly with the increasing number of complications or comorbidities (*p* < 0.05).

### 3.2. Reliability and Validity Test

As shown in Table 3, Bartlett’s test of sphericity yields a chi-square value of 313.033 (*p* < 0.0001) with the KMO index of 0.701. The results of exploratory factor analysis (EFA) demonstrated clean separation of components, with all loadings greater than 0.40 per each item. Five factors were identified in this step as predictors of impact on work, impact on daily life, satisfaction with symptom control and therapy, satisfaction with lifestyle change and self-monitoring, and impact on family and overall health. After combining the opinions of the experts, no items were removed from the original English version during the adaptation and translation process and content and construct validity were established.

During the formal test, the Cronbach’s alpha coefficient for the Chinese brief DQoL was 0.731, suggesting that the scale had an acceptable internal consistency. The Spearman coefficient revealed a low-moderate inverse correlation between DQoL and EQ-5D-5L index and EQ-VAS, respectively (ρ1 = −0.364, *p* < 0.0001; ρ2 = −0.514, *p* < 0.0001). On the whole, the correlation was stronger between DQoL and EQ-VAS than between DQoL and the EQ-5D-5L index. Furthermore, relationships among subgroups were explored in Table 4. Patients with two or more complications tended to achieve the same results of HRQoL from DQoL and EQ-5D-5L index (ρ = −0.561, *p* < 0.0001) while patients who had any risk factors, no complication, or one comorbidity were more likely to get the same results from DQoL and EQ-VAS (ρ < −0.5, *p* < 0.0001). No statistically significant associations were found among other subgroups. The area under the receiver operating characteristic (ROC) curve (AUC) showed a potentially higher predictive capacity of DQoL in patients with age ≥ 60 years, disease duration ≥10 years, any diabetes complications, pneumonia, and arthritis (Supplementary Table S2).

## 4. Discussion

The goal of this study was to develop a Chinese version of the brief DQoL questionnaire to evaluate HRQoL in Chinese T2D patients. It was the first study to validate the 15-item brief version of DQoL for use among the Chinese population. The adaptation and translation was conducted following the guidelines and standards for the translation and cultural adaptation of patient-reported outcome (PRO) measures [15]. Psychometric properties of the scale have been tested on a group of 277 participants in which the content, construct and criterion-related validity and reliability were established.

In the process of carrying out the study, the issue of choosing the appropriate instrument came up first. Although there are many different scales that can be used to describe the HRQoL of people with diabetes, these instruments usually have too many questions and are too complicated to heavily affect compliance. For example, the Diabetes Quality of Life Clinical Trial Questionnaire-Revised (DQLCTQ-R) consists of 57 items; the Diabetes Quality of Life (DQoL) has a total of 46 items; the Diabetes-39 (D-39) has 39 items; the Audit of Diabetes Dependent Quality of Life (ADDQoL) has 19 items. Even though some researchers have tried to reduce items from the original scales, most of them still entail an abundance of questions. Meanwhile, there are a large number of T2D patients in China, quite a few of whom are elderly, with inadequate education and low socioeconomic status, and have poor glycemic control and possibly reduced quality of life [20]. The composition of our recruitment pool has confirmed this fact. Developing a concise and cognitively undemanding scale that conforms to Chinese language habits while retaining its reliability and validity has huge clinical benefits and social significance.

Two measures of validity were conducted in the study besides expert interviews, one for construct validity (EFA) and one for criterion-related validity (Spearman correlation). Five domains were found in which all items of the developed Chinese brief DQoL loaded onto factors at >0.40. The study also revealed a low-moderate inverse correlation between Chinese brief DQoL and EQ-5D-5L (including index and VAS) (ρ1 = −0.364, *p* < 0.0001; ρ2 = −0.514, *p* < 0.0001), which could be reasoned from four levels.

The first reason lies in the different ways to interpret the results. For DQoL, lower score meant better outcomes while for EQ-5D-5L, the opposite was true, which justified the negative correlation between the two questionnaires. Second, EQ-5D-5L was designed as a generic instrument to capture the most general aspects of quality of life that could be used in all patients and enabled a broader comparison across diseases, age and treatments. In comparison, DQoL was a disease-specific instrument and, therefore, usually offered a greater sensitivity in selected populations (i.e., T2D patients). This difference in sensitivity and specificity between the two scales may affect synchronization of the results of the same subject. Additionally, the accuracy of the translation affected the results to some extent as well. The translator should have a deep understanding and professional knowledge of the features and content that the scale aimed to evaluate. In this study, the original English scale underwent forward and back translation by experts as well as editorial review by laypersons to confirm accuracy and appropriateness of wording. Both versions were reviewed for equivalence in English and Chinese for the validation of similarities in language and interpretability, and content were adapted according to the reviewers’ comments [21]. Although the translation was carried out by two experts independently who were fluent in both languages, neither of them was a native speaker. Usage and word choice may still be somewhat inaccurate or inappropriate. Finally yet importantly, cultural and linguistic appropriateness played an essential role. Factors such as a negative attitude towards scientific research and healthcare services, reluctance to disclose personal information, conservative environment that discouraged patients from describing their symptoms, disease stigma, low education level, and the characteristics of target communities or recruitment pool may all lead to cultural bias. An explanation of the study purpose and the informed consent were distributed to patients before enrollment to protect the confidentiality of personally identifiable information (PII). However, we noticed that when DQoL asked questions about sex life and family burden which were often perceived as embarrassing in Asian culture, participants showed reluctance to answer and tended to avoid the question, especially if they were with their families.

Overall, the correlation was stronger between DQoL and EQ-VAS than between DQoL and EQ-5D-5L index, which could be partly addressed by the low education level of participants since a visual analogue scale was easier for them to understand and complete. The highest correlation was observed in patients with two or more complications between DQoL and EQ-5D-5L index (ρ = −0.561, *p* < 0.0001), and in patients with one comorbidity between DQoL and EQ-VAS (ρ = −0.599, *p* < 0.0001). However, there were no statistically significant associations, either between DQoL and the EQ-5D-5L index or between DQoL and EQ-VAS, in patients with two or more comorbidities (*p* > 0.05), which could be explained by the very small sample size (*n* = 10). The Chinese brief DQoL also showed a potentially greater predictive capacity than EQ-5D-5L in patients with specific characteristics.

The limitations of the study should be considered when interpreting the results. First, although the original DQoL questionnaire can be used to identify quality of life issues in patients with type 1 or type 2 diabetes [12], only T2D patients were included in this study. Also, the test-retest reliability was not assessed due to limitations of time and funds. Another reason is that most participants received therapy adjustment and counselling right after the physician–patient encounter. Their opinions were very likely to change after the initial test, especially on diabetes knowledge, satisfaction of current treatment regimen, and frequency of feeling physically ill. Second, the study was based on a sample of patients collected from a single hospital in Shanghai, which may not be representative of the whole diabetes population in China. Further use of this version in national multicenter studies will provide a better understanding of the psychometric properties. Moreover, participants were recruited only from outpatient settings. Although some sample characteristics are consistent with the overall estimate [2,20], the elderly were overrepresented with a mean age of 64 years old. Further research in different clinical settings will add to the credibility of the findings reported in this study, especially in patients with type 1 or type 2 diabetes of all ages.

## 5. Conclusions

This study suggested that the simplified Chinese version of the brief DQoL questionnaire developed was a potentially appropriate tool to assess quality of life in Chinese T2D patients. Future research is needed to further evaluate the instrument to support its application in China. Cultural and linguistic differences should also be emphasized in the translation and adaptation process.

## Figures and Tables

**Table 1:** Demographic and clinical profiles of participants (*n* = 277).

Demographics		Frequency (*n*)	Percent (%)	Mean (SD)
Gender	Male	159	57.4	
	Female	118	42.6	
Age (Years)	<60	81	29.2	64.0 (9.7)
	≥60	196	70.8	
BMI (kg/m^2^)	<25	167	60.3	24.60 (3.78)
	≥25	110	39.7	
Education Level	Primary school or below	104	37.5	
	Secondary school	95	34.3	
	High school	54	19.5	
	Bachelor or above	24	8.7	
Occupation	Employed	36	13	
	Unemployed	2	0.7	
	Self-employed	5	1.8	
	Retired	234	84.5	
Duration of Disease (Years)	<10	101	36.5	12.3 (8.0)
	≥10	176	63.5	
Fasting Plasma Glucose (mmol/L)	<7	105	37.9	8.48 (6.37)
	≥7	172	62.1	
Diabetes Complications	Diabetic retinopathy	99	35.7	
	Autonomic neuropathy	86	31	
	Dermopathy	68	24.5	
	Cardiovascular disease	40	14.4	
	Cerebrovascular disease	22	7.9	
	Peripheral neuropathy	18	6.5	
	Diabetic foot	1	0.4	
No. of Diabetes Complications	0	123	44.4	1.2
	1	66	23.8	(1.5)
	≥2	88	31.8	
Comorbidities	Pneumonia	21	7.6	
	Arthritis	42	15.2	
	Anxiety/Depression	3	1.1	
	Cancer	1	0.4	
	Musculoskeletal disease	3	1.1	
No. of Comorbidities	0	214	77.3	0.3
	1	53	19.1	(0.5)
	≥2	10	3.6	

**Table 2:** Comparison of scores of Chinese brief Diabetes Quality of Life (DQoL), 5-level version of EuroQoL-5 (EQ-5D-5L) index, and EQ-VAS in subgroups.

Variables		*n*	DQoL		EQ-5D-5L Index		EQ-VAS	
			Median	*p*	Median	*p*	Median	*p*
BMI (kg/m^2^)	<25	167	28	0.432	0.94	0.579	70	0.380
	≥25	110	28		0.94		75	
Age (Years)	<60	81	27	0.479	1.00	<0.0001	80	0.009
	≥60	196	28		0.90		70	
Duration (Years)	<10	101	26	0.001	0.94	0.036	80	0.002
	≥10	176	29		0.94		70	
FPG (mmol/L)	<7	105	28	0.744	0.94	0.578	78	0.012
	≥7	172	28		0.94		70	
No. of Diabetes Complications	0	123	26	<0.0001	1.00	<0.0001	80	<0.0001
	1	66	28		0.94		75	
	≥2	88	30		0.89		70	
No. of Comorbidities	0	214	28	0.004	0.94	<0.0001	75	0.004
	1	53	28		0.89		70	
	≥2	10	34		0.75		65	

**Table 3:** The results of exploratory factor analysis of the Chinese brief DQoL.

Items		Factor Loadings				
		1	2	3	4	5
1. How often do you worry about whether you will miss work?		0.852	−0.005	0.134	−0.059	0.008
2. How often do you feel diabetes limits your career?		0.833	0.174	0.079	0.077	−0.030
3. How satisfied are you with the amount of time it takes to manage your diabetes?		0.489	0.066	−0.373	0.397	0.245
4. How often do you have a bad night’s sleep because of diabetes?		0.063	0.818	−0.127	−0.068	0.079
5. How satisfied are you with your sex life?		0.071	0.798	0.004	0.225	−0.001
6. How satisfied are you with your knowledge about your diabetes?		0.125	0.481	0.210	0.114	0.378
7. How often do you worry about whether you will pass out?		0.200	0.010	0.754	0.212	0.093
8. How often do you have pain because of the treatment for your diabetes?		0.266	0.189	0.603	0.036	0.015
9. How satisfied are you with time spent getting checkups for your diabetes?		0.200	0.342	−0.563	0.176	0.081
10. How satisfied are you with your current diabetes treatment?		0.255	0.151	−0.498	0.330	0.321
11. How often do you find that you eat something you shouldn’t rather than tell someone that you have diabetes?		−0.088	−0.025	0.072	0.761	−0.198
12. How satisfied are you with the time you spend exercising?		0.058	0.187	−0.073	0.672	0.240
13. How satisfied are you with the time it takes to determine your sugar level?		0.395	0.095	0.101	0.515	0.279
14. How satisfied are you with the burden your diabetes is placing on your family?		0.081	−0.107	−0.143	0.177	0.819
15. How often do you feel physically ill?		−0.088	0.351	0.074	−0.100	0.742
Cumulative % of Variance (Rotated)		13.874	26.721	38.498	50.229	61.725
Kaiser–Meyer–Olkin Measure of Sampling Adequacy						0.701
Bartlett’s Test of Sphericity	Approx. Chi-Square					313.033
	df					105.000
	Sig.					<0.0001

**Table 4:** Correlation between DQoL scores and EQ-5D-5L index and EQ-VAS in subgroups.

Variables		*n*	EQ-5D-5L Index		EQ-VAS	
			ρ	*p*	ρ	*p*
Overall		277	−0.364	<0.0001	−0.514	<0.0001
BMI (kg/m^2^)	<25	167	−0.380	<0.0001	−0.485	<0.0001
	≥25	110	−0.349	<0.0001	−0.593	<0.0001
Age (Years)	<60	81	−0.274	0.013	−0.520	<0.0001
	≥60	196	−0.421	<0.0001	−0.520	<0.0001
Duration (Years)	<10	101	−0.225	0.024	−0.468	<0.0001
	≥10	176	−0.419	<0.0001	−0.510	<0.0001
FPG (mmol/L)	<7	105	−0.379	<0.0001	−0.454	<0.0001
	≥7	172	−0.363	<0.0001	−0.566	<0.0001
No. of Diabetes Complications	0	123	−0.200	0.026	−0.574	<0.0001
	1	66	−0.276	0.025	−0.389	0.001
	≥2	88	−0.561	<0.0001	−0.433	<0.0001
No. of Comorbidities	0	214	−0.394	<0.0001	−0.482	<0.0001
	1	53	−0.081	0.566	−0.599	<0.0001
	≥2	10	−0.165	0.649	−0.527	0.117

## Supplementary Materials

The following are available online at https://www.mdpi.com/1660-4601/17/23/8792/s1, Table S1: Tests of normality and histogram of distribution among subgroups, Table S2: Comparison of AUC values of DQoL and EQ-5D-5L.
