# Supplementary material for: Validation of the Simplified Chinese Version of the Brief Diabetes Quality of Life (DQoL) Questionnaire Based on a Cross-Sectional Study
Source: Int J Environ Res Public Health. 2020 Nov 26;17(23):8792. doi: 10.3390/ijerph17238792 (PMC7729895; doi:10.3390/ijerph17238792)

**Table S1. Tests of normality and histogram of distribution among subgroups**

S1a. Tests of normality and histogram of distribution based on BMI (1 = “BMI<25 kg/m^2^”, 2 = “BMI≥25 kg/m^2^”)

| **Tests of Normality** | | | | | | | |
| --- | --- | --- | --- | --- | --- | --- | --- |
|  | BMI_level | Kolmogorov-Smirnov^a^ | | | Shapiro-Wilk | | |
|  |  | Statistic | df | Sig. | Statistic | df | Sig. |
| DQoL | 1 | 0.120 | 167 | <0.0001 | 0.944 | 167 | <0.0001 |
|  | 2 | 0.120 | 110 | <0.0001 | 0.970 | 110 | 0.014 |
| EQ_Index | 1 | 0.243 | 167 | <0.0001 | 0.672 | 167 | <0.0001 |
|  | 2 | 0.202 | 110 | <0.0001 | 0.795 | 110 | <0.0001 |
| EQ_VAS | 1 | 0.199 | 167 | <0.0001 | 0.836 | 167 | <0.0001 |
|  | 2 | 0.181 | 110 | <0.0001 | 0.919 | 110 | <0.0001 |
| a. Lilliefors Significance Correction | | | | | | | |


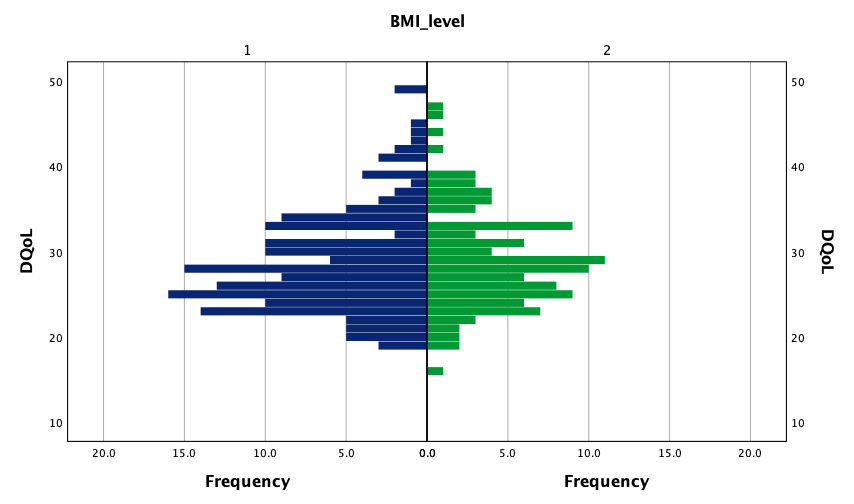


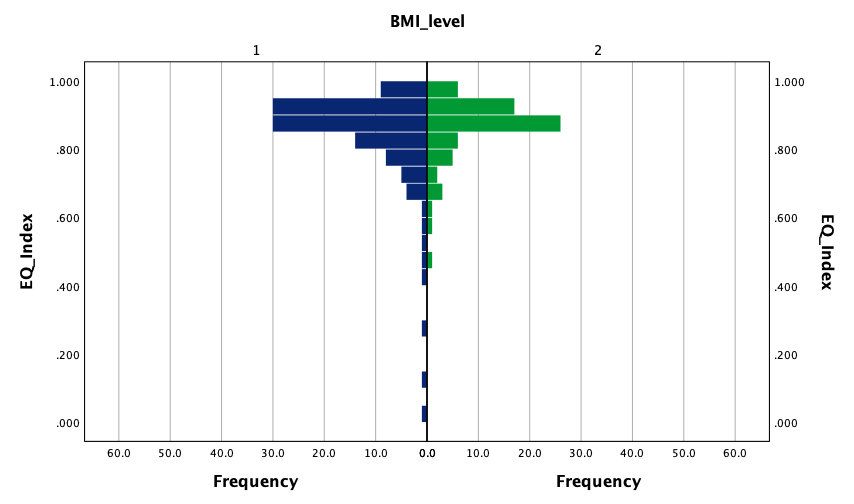


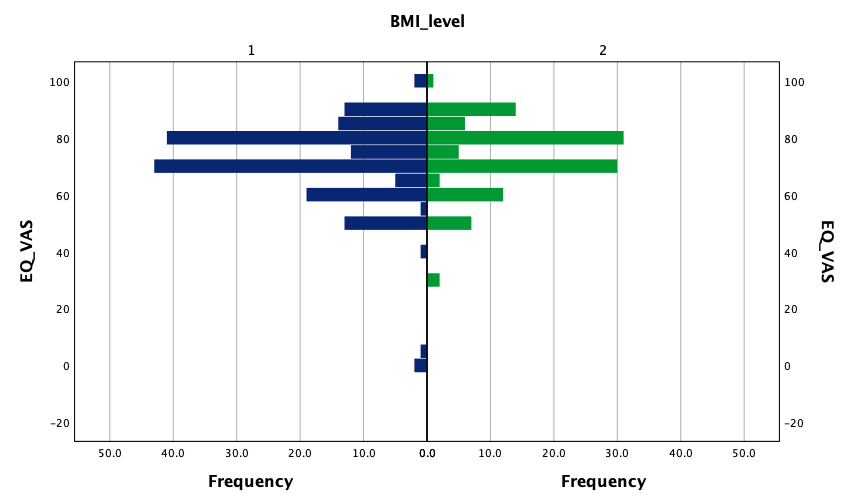


S1b. Tests of normality and histogram of distribution based on age (1 = “age<60 years”, 2 = “age≥60 years”)

| **Tests of Normality** | | | | | | | |
| --- | --- | --- | --- | --- | --- | --- | --- |
|  | Age_level | Kolmogorov-Smirnov^a^ | | | Shapiro-Wilk | | |
|  |  | Statistic | df | Sig. | Statistic | df | Sig. |
| DQoL | 1 | 0.160 | 81 | <0.0001 | 0.909 | 81 | <0.0001 |
|  | 2 | 0.098 | 196 | <0.0001 | 0.971 | 196 | <0.0001 |
| EQ_Index | 1 | 0.311 | 81 | <0.0001 | 0.545 | 81 | <0.0001 |
|  | 2 | 0.217 | 196 | <0.0001 | 0.720 | 196 | <0.0001 |
| EQ_VAS | 1 | 0.184 | 81 | <0.0001 | 0.941 | 81 | 0.001 |
|  | 2 | 0.219 | 196 | <0.0001 | 0.846 | 196 | <0.0001 |
| a. Lilliefors Significance Correction | | | | | | | |


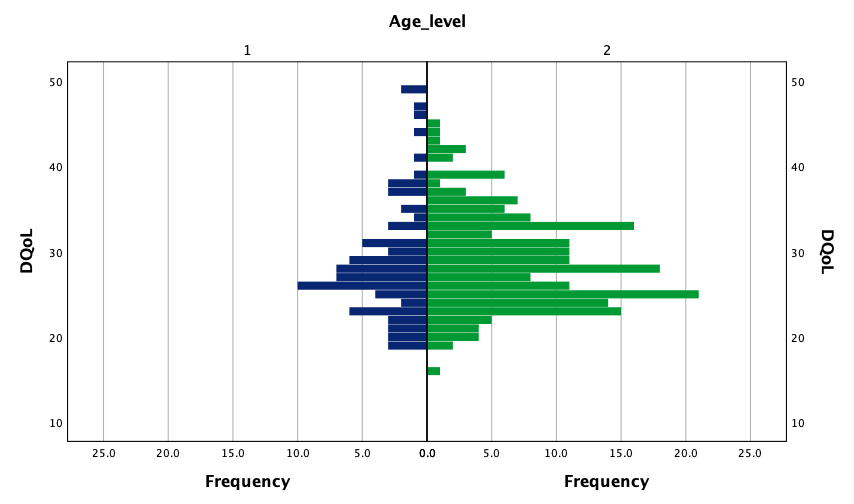


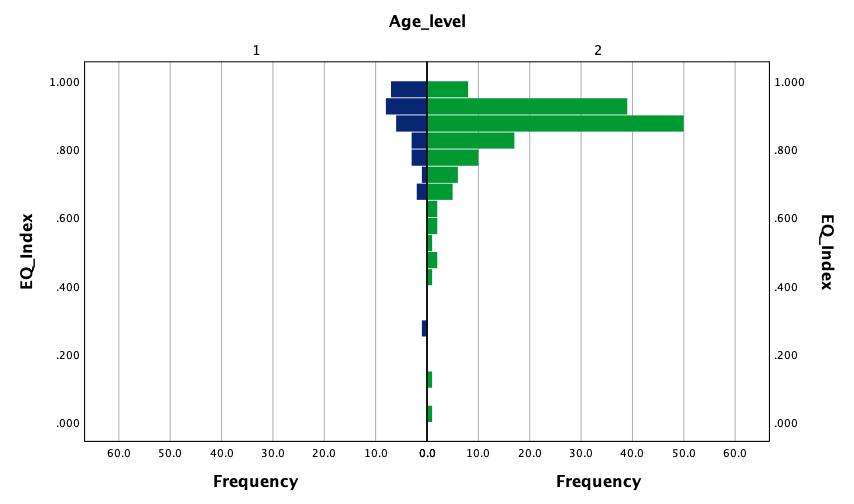


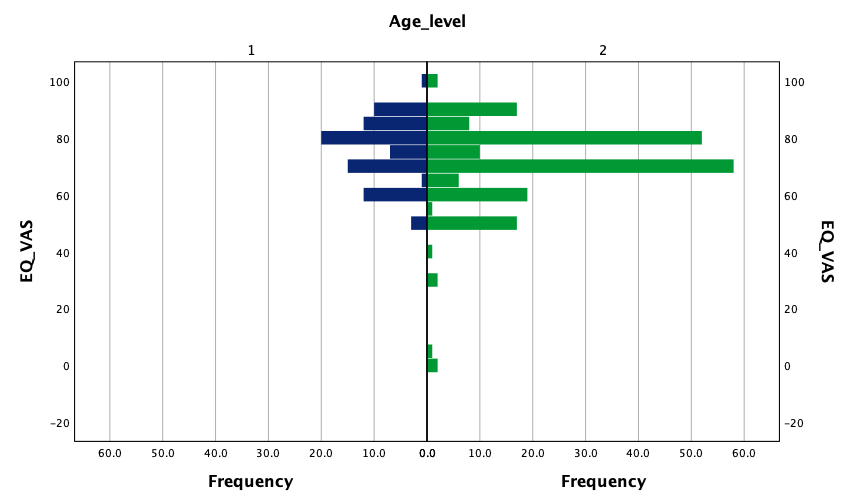


S1c. Tests of normality and histogram of distribution based on disease duration (1 = “duration<10 years”, 2 = “duration≥10 years”)

| **Tests of Normality** | | | | | | | |
| --- | --- | --- | --- | --- | --- | --- | --- |
|  | duration_level | Kolmogorov-Smirnov^a^ | | | Shapiro-Wilk | | |
|  |  | Statistic | df | Sig. | Statistic | df | Sig. |
| DQoL | 1 | 0.150 | 101 | <0.0001 | 0.902 | 101 | <0.0001 |
|  | 2 | 0.097 | 176 | <0.0001 | 0.974 | 176 | 0.002 |
| EQ_Index | 1 | 0.234 | 101 | <0.0001 | 0.678 | 101 | <0.0001 |
|  | 2 | 0.226 | 176 | <0.0001 | 0.715 | 176 | <0.0001 |
| EQ_VAS | 1 | 0.161 | 101 | <0.0001 | 0.916 | 101 | <0.0001 |
|  | 2 | 0.196 | 176 | <0.0001 | 0.852 | 176 | <0.0001 |
| a. Lilliefors Significance Correction | | | | | | | |


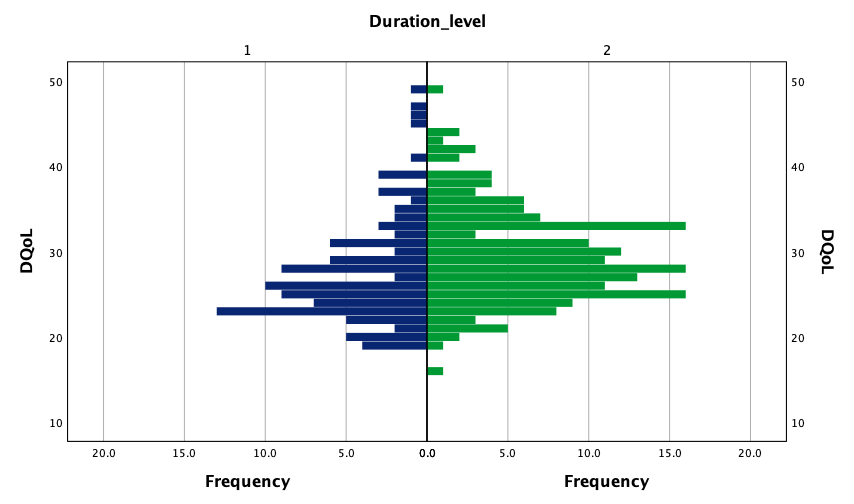


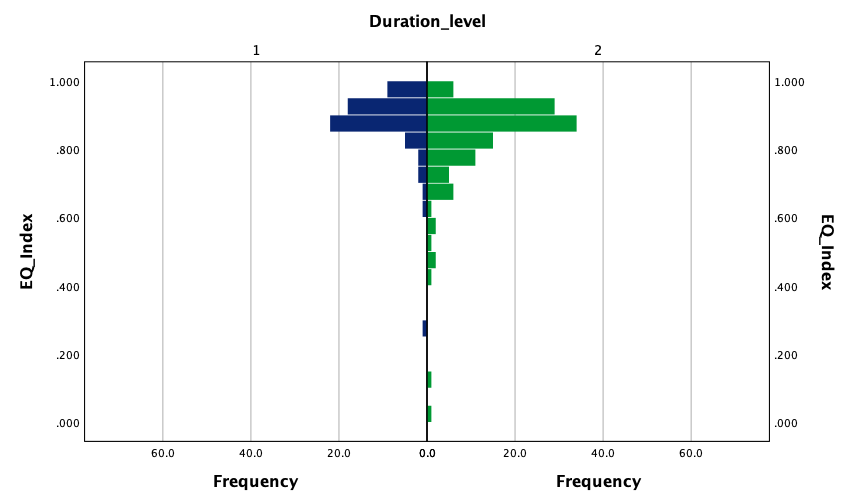


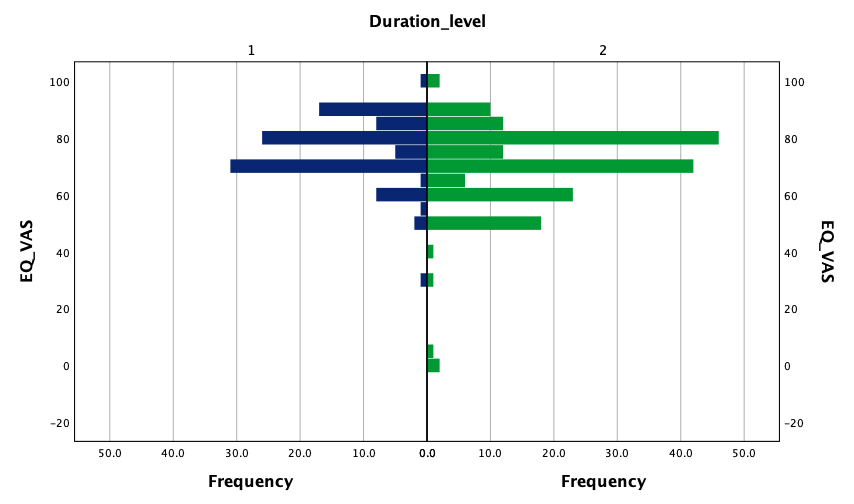


S1d. Tests of normality and histogram of distribution based on FPG (1 = “FPG<7 mmol/L”, 2 = “FPG≥7 mmol/L”)

| **Tests of Normality** | | | | | | | |
| --- | --- | --- | --- | --- | --- | --- | --- |
|  | FPG_level | Kolmogorov-Smirnov^a^ | | | Shapiro-Wilk | | |
|  |  | Statistic | df | Sig. | Statistic | df | Sig. |
| DQoL | 1 | 0.127 | 105 | <0.0001 | 0.956 | 105 | 0.002 |
|  | 2 | 0.093 | 172 | 0.001 | 0.956 | 172 | <0.0001 |
| EQ_Index | 1 | 0.179 | 105 | <0.0001 | 0.843 | 105 | <0.0001 |
|  | 2 | 0.243 | 172 | <0.0001 | 0.689 | 172 | <0.0001 |
| EQ_VAS | 1 | 0.186 | 105 | <0.0001 | 0.916 | 105 | <0.0001 |
|  | 2 | 0.173 | 172 | <0.0001 | 0.872 | 172 | <0.0001 |
| a. Lilliefors Significance Correction | | | | | | | |


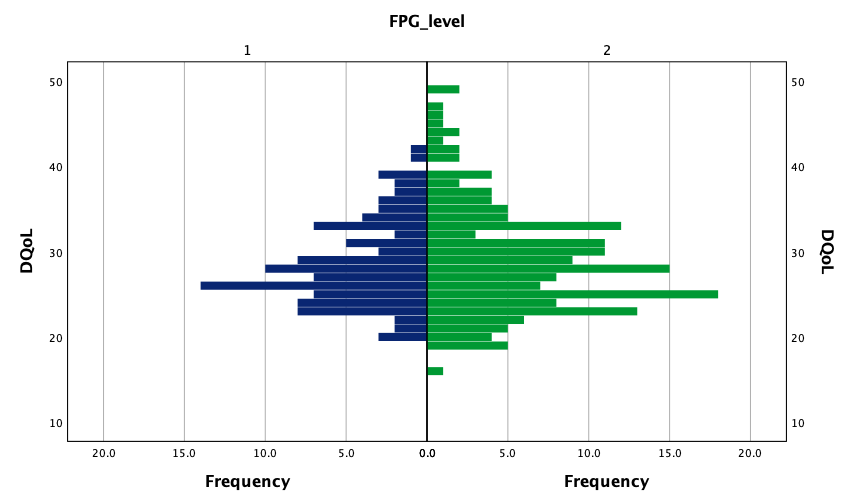


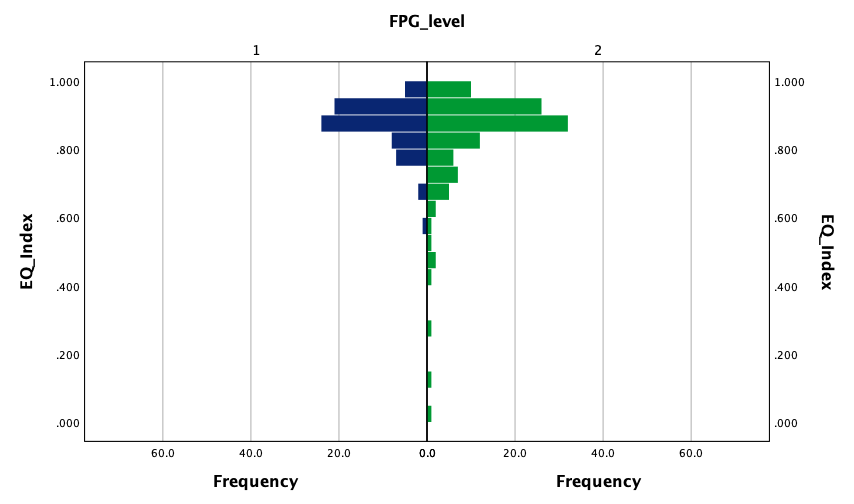


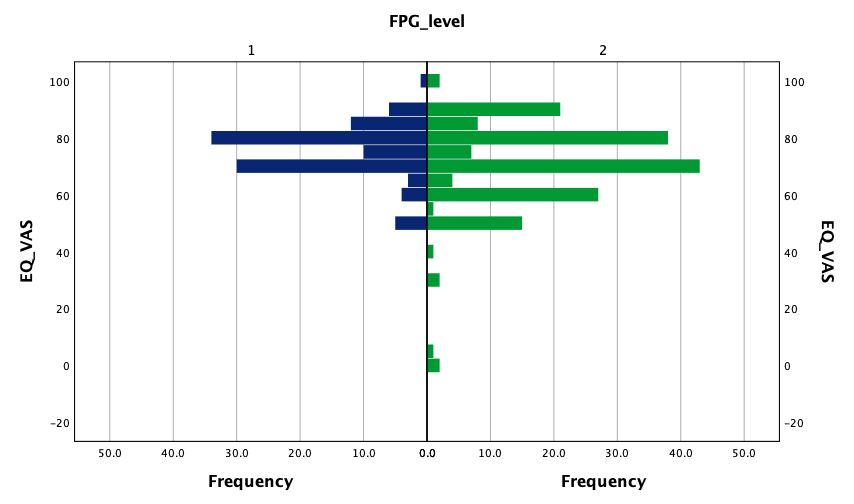


S1e. Tests of normality and histogram of distribution based on number of complications (0 = “0”, 1 = “1” 2= “≥2”)

| **Tests of Normality** | | | | | | | |
| --- | --- | --- | --- | --- | --- | --- | --- |
|  | Comp_level | Kolmogorov-Smirnov^a^ | | | Shapiro-Wilk | | |
|  |  | Statistic | df | Sig. | Statistic | df | Sig. |
| DQoL | 0 | 0.152 | 123 | <0.0001 | 0.930 | 123 | <0.0001 |
|  | 1 | 0.116 | 66 | 0.028 | 0.926 | 66 | 0.001 |
|  | 2 | 0.074 | 88 | 0.200* | 0.973 | 88 | 0.059 |
| EQ_Index | 0 | 0.281 | 123 | <0.0001 | 0.736 | 123 | <0.0001 |
|  | 1 | 0.188 | 66 | <0.0001 | 0.799 | 66 | <0.0001 |
|  | 2 | 0.203 | 88 | <0.0001 | 0.748 | 88 | <0.0001 |
| EQ_VAS | 0 | 0.193 | 123 | <0.0001 | 0.803 | 123 | <0.0001 |
|  | 1 | 0.162 | 66 | <0.0001 | 0.945 | 66 | 0.006 |
|  | 2 | 0.231 | 88 | <0.0001 | 0.826 | 88 | <0.0001 |
| *. This is a lower bound of the true significance.  a. Lilliefors Significance Correction | | | | | | | |


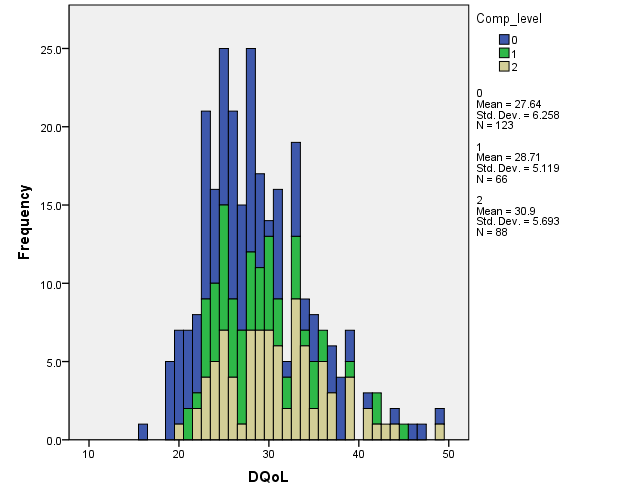


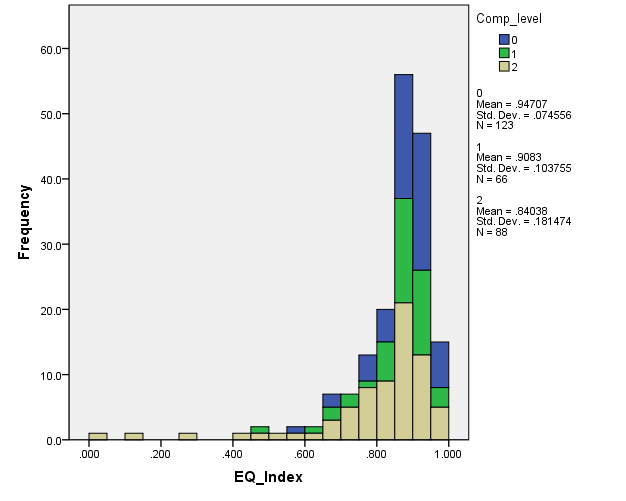


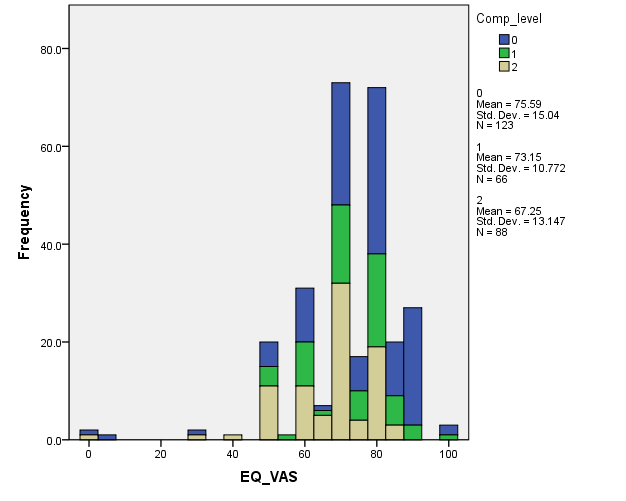


S1f. Tests of normality and histogram of distribution based on number of comorbidities

| **Tests of Normality** | | | | | | | |
| --- | --- | --- | --- | --- | --- | --- | --- |
|  | Comorbidities | Kolmogorov-Smirnov^a^ | | | Shapiro-Wilk | | |
|  |  | Statistic | df | Sig. | Statistic | df | Sig. |
| DQoL | 0 | 0.101 | 214 | <0.0001 | 0.955 | 214 | <0.0001 |
|  | 1 | 0.141 | 53 | 0.011 | 0.941 | 53 | 0.011 |
|  | 2 | 0.175 | 10 | 0.200* | 0.942 | 10 | 0.580 |
| EQ_Index | 0 | 0.257 | 214 | <0.0001 | 0.654 | 214 | <0.0001 |
|  | 1 | 0.242 | 53 | <0.0001 | 0.825 | 53 | <0.0001 |
|  | 2 | 0.220 | 10 | 0.185 | 0.845 | 10 | 0.050 |
| EQ_VAS | 0 | 0.174 | 214 | <0.0001 | 0.899 | 214 | <0.0001 |
|  | 1 | 0.233 | 53 | <0.0001 | 0.741 | 53 | <0.0001 |
|  | 2 | 0.254 | 10 | 0.066 | 0.841 | 10 | 0.045 |
| *. This is a lower bound of the true significance.  a. Lilliefors Significance Correction | | | | | | | |


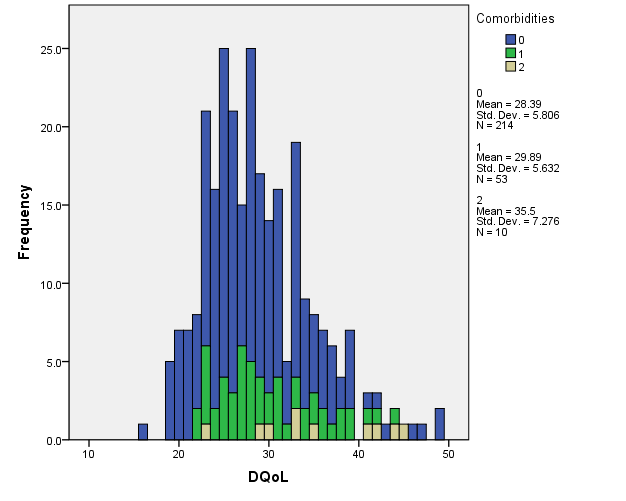


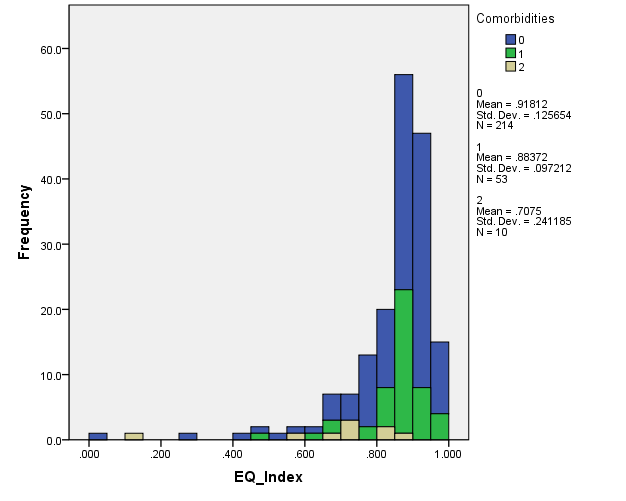


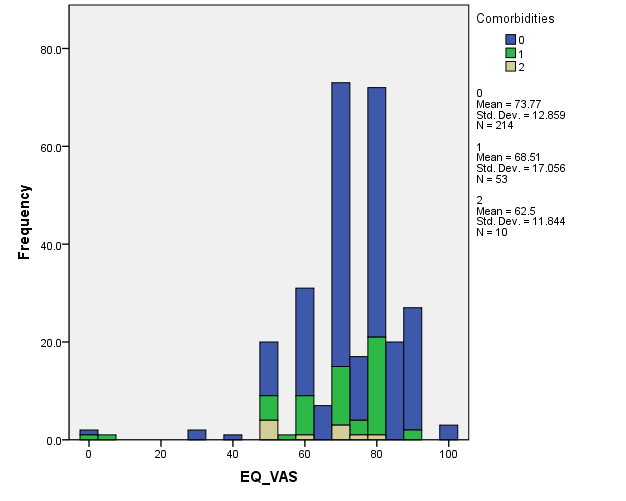

Supplement: Supplementary file 1 [file ijerph-17-08792-s001.zip › Supplementary 1.docx]
