# Supplementary material for: Validation of the Simplified Chinese Version of the Brief Diabetes Quality of Life (DQoL) Questionnaire Based on a Cross-Sectional Study
Source: Int J Environ Res Public Health. 2020 Nov 26;17(23):8792. doi: 10.3390/ijerph17238792 (PMC7729895; doi:10.3390/ijerph17238792)
Supplement: Supplementary file 1 [file ijerph-17-08792-s001.zip › Supplementary 2.docx]

**Table S2. Comparison of AUC values of DQoL and EQ-5D-5L**

| **Variables** | **N** | **DQoL** | |  | **EQ-5D-5L** | |
| --- | --- | --- | --- | --- | --- | --- |
|  |  | **AUC** | **95% CI** |  | **AUC** | **95% CI** |
| BMI≥25 kg/m^2^ | 110 | 0.504 | (0.428, 0.580) | 0.568 | | (0.494, 0.643) |
| Age≥60 years | 196 | 0.527 | (0.450, 0.604) | 0.297 | | (0.228, 0.365) |
| Disease duration≥10 years | 176 | 0.621 | (0.550, 0.692) | 0.427 | | (0.358, 0.495) |
| FPG≥7 mmol/L | 172 | 0.512 | (0.443, 0.580) | 0.481 | | (0.412, 0.549) |
| **Comorbidities** | | | | | | |
| Pneumonia | 21 | 0.659 | (0.533, 0.786) | 0.369 | | (0.252, 0.486) |
| Arthritis | 42 | 0.573 | (0.477, 0.669) | 0.271 | | (0.198, 0.344) |
| Anxiety/Depression | 3 | 0.731 | (0.526, 0.937) | 0.245 | | (0.000, 0.535) |
| Cancer | 1 | 0.938 | (0.907, 0.969) | 0.819 | | (0.609, 1.000) |
| Musculoskeletal disease | 3 | 0.743 | (0.529, 0.958) | 0.308 | | (0.000, 0.722) |
| **Diabetes complications** | | | | | | |
| Diabetic retinopathy | 99 | 0.626 | (0.560, 0.691) | 0.359 | | (0.291, 0.426) |
| Autonomic neuropathy | 86 | 0.613 | (0.543, 0.683) | 0.293 | | (0.226, 0.360) |
| Dermopathy | 68 | 0.621 | (0.548, 0.693) | 0.351 | | (0.275, 0.426) |
| Cardiovascular disease | 40 | 0.697 | (0.602, 0.792) | 0.267 | | (0.178, 0.356) |
| Cerebrovascular disease | 22 | 0.788 | (0.689, 0.887) | 0.208 | | (0.090, 0.326) |
| Peripheral neuropathy | 18 | 0.767 | (0.631, 0.903) | 0.187 | | (0.062, 0.312) |
| Diabetic foot | 1 | 0.877 | (0.836, 0.917) | 0.112 | | (0.075, 0.150) |
